# Dissociation of Cohesin from Chromosome Arms and Loss of Arm Cohesion during Early Mitosis Depends on Phosphorylation of SA2

**DOI:** 10.1371/journal.pbio.0030069

**Published:** 2005-03-01

**Authors:** Silke Hauf, Elisabeth Roitinger, Birgit Koch, Christina M Dittrich, Karl Mechtler, Jan-Michael Peters

**Affiliations:** **1**Research Institute of Molecular PathologyViennaAustria; Stowers Institute for Medical ResearchUnited States of America

## Abstract

Cohesin is a protein complex that is required to hold sister chromatids together. Cleavage of the Scc1 subunit of cohesin by the protease separase releases the complex from chromosomes and thereby enables the separation of sister chromatids in anaphase. In vertebrate cells, the bulk of cohesin dissociates from chromosome arms already during prophase and prometaphase without cleavage of Scc1. Polo-like kinase 1 (Plk1) and Aurora-B are required for this dissociation process, and Plk1 can phosphorylate the cohesin subunits Scc1 and SA2 in vitro, consistent with the possibility that cohesin phosphorylation by Plk1 triggers the dissociation of cohesin from chromosome arms. However, this hypothesis has not been tested yet, and in budding yeast it has been found that phosphorylation of Scc1 by the Polo-like kinase Cdc5 enhances the cleavability of cohesin, but does not lead to separase-independent dissociation of cohesin from chromosomes. To address the functional significance of cohesin phosphorylation in human cells, we have searched for phosphorylation sites on all four subunits of cohesin by mass spectrometry. We have identified numerous mitosis-specific sites on Scc1 and SA2, mutated them, and expressed nonphosphorylatable forms of both proteins stably at physiological levels in human cells. The analysis of these cells lines, in conjunction with biochemical experiments in vitro, indicate that Scc1 phosphorylation is dispensable for cohesin dissociation from chromosomes in early mitosis but enhances the cleavability of Scc1 by separase. In contrast, our data reveal that phosphorylation of SA2 is essential for cohesin dissociation during prophase and prometaphase, but is not required for cohesin cleavage by separase. The similarity of the phenotype obtained after expression of nonphosphorylatable SA2 in human cells to that seen after the depletion of Plk1 suggests that SA2 is the critical target of Plk1 in the cohesin dissociation pathway.

## Abstract 2

Cohesin holds newly replicated chromosomes together until a cell is ready to divide. These authors show how phosphorylation regulates cohesin function

## Introduction

Faithful inheritance of the genome depends on its accurate replication and correct distribution to the two daughter cells. In eukaryotes, the two copies of a chromosome that are generated in S-phase (sister chromatids) remain connected until they are separated in anaphase of mitosis. This physical association (cohesion) allows the mitotic segregation machinery to handle sister chromatids as entities that have to be distributed to opposite poles. Sister chromatid cohesion depends on cohesin, a protein complex that is highly conserved in evolution and consists of at least four subunits: two “structural maintenance of chromosomes” proteins, Smc1 and Smc3, the so-called “kleisin” subunit Scc1 (also called Rad21 or Mcd1), and Scc3 (reviewed in [1]). Cells of humans, *Xenopus,* and other higher eukaryotes contain two mitotic orthologs of Scc3, called SA1 and SA2. Cohesin complexes in these cells contain either SA1 or SA2, but not both [2,3].

In order to segregate sister chromatids to opposite poles in anaphase, cohesin has to be removed from chromosomes. In budding yeast, the prevalent mode of cohesin removal is by proteolytic cleavage of the Scc1 subunit at the onset of anaphase by the endopeptidase separase [4,5]. Prior to anaphase, separase is kept inactive by its inhibitor securin [5,6,7,8,9,10], and in vertebrate cells also by inhibitory phosphorylation mediated by Cdk1 [11]. Both securin and Cdk1's activating subunit cyclin B are ubiquitinated at the onset of anaphase by the anaphase-promoting complex/cyclosome, leading to their proteasome-dependent degradation and to separase activation (reviewed in [12]).

In higher eukaryotes, removal of cohesin from chromosomes occurs in at least two steps. During prophase and prometaphase, the bulk of cohesin dissociates from chromosomes without Scc1 cleavage [3,13]. Only minor amounts of cohesin (an estimated 10%) remain on chromosomes up to metaphase, preferentially at centromeres [10,14]. A similarly minor amount of cohesin, presumably the chromosome-bound fraction, is cleaved by separase at the onset of anaphase [10]. As in budding yeast, the cleavage of Scc1 is essential for anaphase to occur [15]. Two mitosis-specific protein kinases are required for the cleavage-independent removal of cohesin from chromosome arms: Plk1, called Plx1 in *Xenopus,* and Aurora-B [16,17,18]. Plk1/Plx1, but not Aurora-B, can phosphorylate the cohesin subunits Scc1 and SA2 in vitro [16,17], and is required for their phosphorylation in *Xenopus* egg extracts [16]. In these extracts, the ability of cohesin to bind to chromatin correlates inversely with its phosphorylation state [16]. This observation, and the finding that Plk1 is required for cohesin dissociation from chromosomes, raise the possibility that phosphorylation of cohesin by Plk1 leads to its cleavage-independent dissociation from chromosomes. However, it is unknown whether Plk1's critical target in the cohesin dissociation process is cohesin itself, and whether cohesin phosphorylation is required for dissociation of the complex from chromosomes. So far, the functional relevance of cohesin phosphorylation has been studied only in budding yeast. In this organism, Scc1 is also phosphorylated by a Polo-like kinase, called Cdc5, but this modification does not seem to result in cohesin's dissociation from chromatin; rather, it renders Scc1 more susceptible to cleavage by separase [19,20].

To test whether cohesin phosphorylation is required for its dissociation from chromosome arms during prophase and prometaphase, and to address whether this modification could explain the requirement for Plk1 in this process, we have searched for phosphorylation sites on all four subunits of the human cohesin complex by mass spectrometry. We have identified numerous mitosis-specific sites on Scc1 and SA2, mutated them, and expressed wild-type and nonphosphorylatable forms of both proteins stably at physiological levels in cultured human cells. The analysis of these cell lines, in conjunction with biochemical experiments in vitro, imply that Scc1 phosphorylation is dispensable for cohesin dissociation from chromosomes in early mitosis, but enhances the cleavability of Scc1 by separase. In contrast, our data reveal that phosphorylation of SA2 is essential for cohesin dissociation during prophase and prometaphase, but is not required for cohesin cleavage by separase. The similarity of the phenotype obtained after expression of nonphosphorylatable SA2 (this study) to that seen after the depletion of Plk1 [18] strongly suggests that SA2 is the critical target of Plk1 in the cohesin dissociation pathway.

## Results

### Identification of Mitosis-Specific Phosphorylation Sites on Human Cohesin

**Figure 1:**
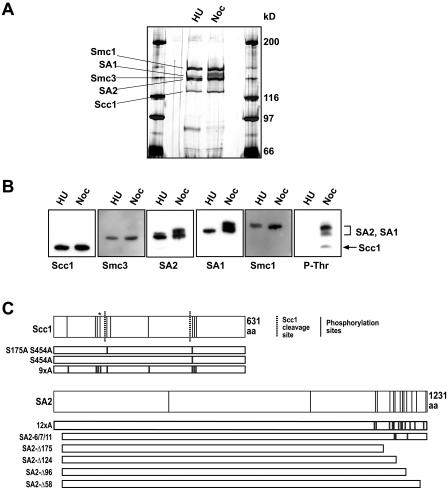
Identification of Mitosis-Specific Phosphorylation Sites on Human Cohesin (A and B) Cohesin was immunoprecipitated by antibody 447 (which recognizes SA1 and SA2) from extracts prepared from HeLa cells that were either arrested in S-phase by hydroxyurea (HU) or in mitosis by nocodazole (Noc). Cohesin was eluted by buffer of low pH and analyzed by (A) silver staining and (B) immunoblotting with antibodies to cohesin subunits and phosphorylated threonine (P-Thr). (C) Schematic representation of the phosphorylation sites on Scc1 and SA2 that were identified by mass spectrometry, and of the mutant versions of the proteins that have been generated. The star indicates a phosphorylation site that was found in both interphase and mitotic Scc1. All SA2 constructs used for in vitro experiments lack the 69 N-terminal amino acids. SA2-WT-myc and SA2–12xA-myc cell lines contain the entire open reading frame of 1,231 amino acids.

The SA1, SA2, and Scc1 subunits of vertebrate cohesin complexes are phosphorylated specifically in mitosis [2,14,16]. To be able to analyze the functional relevance of these modifications, we mapped mitosis-specific phosphorylation sites on human cohesin by mass spectrometry. We prepared lysates from HeLa cells that had been arrested either at the G1/S transition (interphase) by hydroxyurea or in mitosis by the spindle poison nocodazole, and immunoprecipitated SA1- and SA2-containing cohesin complexes with antibody 447, which recognizes the C termini of both SA1 and SA2 [3]. Separation of the isolated proteins by SDS-PAGE and staining with silver (Figure 1A) or cohesin-specific antibodies (Figure 1B) demonstrated reduced electrophoretic mobility of both SA1 and SA2 when the complexes had been isolated from mitotic cells, indicating that the mitosis-specific phosphorylation of these subunits was preserved during the isolation procedure. This notion was confirmed by immunoblotting with phosphothreonine-specific antibodies. This assay yielded a pattern that was consistent with the presence of phosphothreonine residues on SA1, SA2, and Scc1 in mitosis, whereas no phosphothreonine signal was detected on cohesin subunits isolated from interphase cells (Figure 1B).

To identify phosphorylation sites, purified cohesin complexes were digested with various proteases in solution and analyzed by high performance liquid chromatography-electrospray ionization-tandem mass spectrometry (HPLC-ESI-MS/MS). In total, 28 phosphorylated serine or threonine residues were identified. Of these, 18 could be assigned unequivocally to specific residues, whereas in ten cases it could not be determined which of several serine and threonine residues in a given peptide was phosphorylated (Figure 1C and Table 1). Two sites each were found in Smc1 and Smc3, ten in Scc1, and 14 in SA2. We were unable to identify phosphorylated peptides derived from SA1, presumably because SA1-containing cohesin complexes are much less abundant than SA2-containing complexes [2,3]. By analyzing cohesin peptides obtained by digestion with various proteases, we were able to obtain a sequence coverage of approximately 80% for each of the subunits (Figure S1 and unpublished data). This indicates that our analysis was theoretically able to identify the majority of in vivo phosphorylation sites on cohesin subunits, although we cannot exclude the possibility that some sites have gone undetected.

**Table 1:**
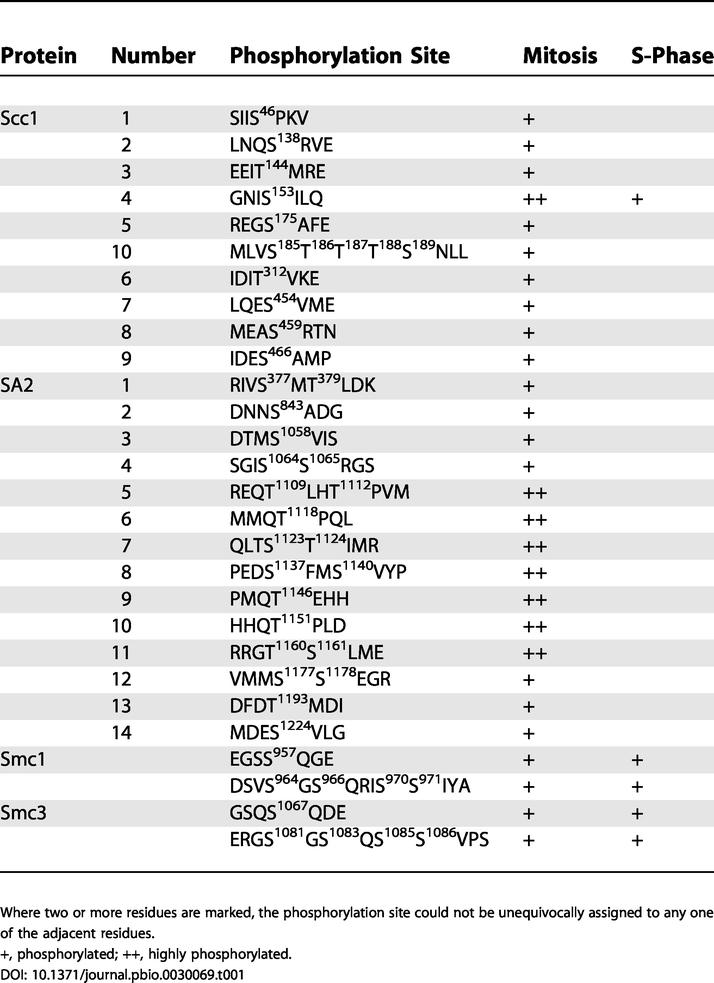
Phosphorylation Sites Identified in Cohesin Subunits

Where two or more residues are marked, the phosphorylation site could not be unequivocally assigned to any one of the adjacent residues

+, phosphorylated; ++, highly phosphorylated

DOI: 10.1371/journal.pbio.0030069.t001

The phosphorylation sites we identified on Smc1 and Smc3 were present in complexes from both interphase and mitotic cells, and we therefore did not analyze them any further. Two of these sites (Ser^966^ and Ser^957^ in Smc1) have been shown to be phosphorylated by the ATM kinase in response to DNA damage [21,22]. Only one of the sites in Scc1 (Ser^153^), and none in SA2, was found phosphorylated in interphase. All other sites were only identified in cohesin samples from mitotic cells. These results confirm that Scc1 and SA1/2 are specifically phosphorylated in mitosis.

Since Plk1/Plx1 can phosphorylate Scc1 and SA1/SA2 in vitro and is required for the phosphorylation of these subunits in *Xenopus* egg extracts [16], we compared the sequence surrounding the identified sites to the consensus sequences that have been reported as binding and phosphorylation sites for Plk1. The C-terminal Polo box domain of Plk1 binds to phosphorylation sites for which the consensus S-[pT/pS]-[P/x] has been proposed [23]. These phosphorylation sites are thought to be generated by proline-directed kinases, such as Cdk1, that “prime” the substrate for subsequent recognition by Plk1. Once bound to the substrate, Plk1 is thought to phosphorylate sites that are distinct from the one that is recognized by the Polo box. Based on in vitro phosphorylation experiments using peptides derived from the phosphatase Cdc25C, Nakajima et al. [24] have proposed the consensus (E/D)-x-(S/T)Φ-x-(E/D) for Plk1 phosphorylation-sites, where Φ signifies a hydrophobic amino acid, whereas Barr et al. [25] have suggested the consensus (E/D/Q)-x-(S/T)Φ. One putative phosphorylation site in Scc1 (Thr^186^) and three putative sites in SA2 (Ser^1065^, Thr^1124^, and Ser^1178^) match the Polo box-binding consensus sequence, although none of these sites contains a proline residue at the +1 position (these are putative sites because they are present in peptides in which the exact identity of the residue carrying the phosphomoiety could not be determined; Table 1). We also note that, although Cdk1 can phosphorylate SA1/2 in vitro [2], it does not appear to be essential for cohesin dissociation in human cells and *Xenopus* egg extracts [3,16]. Two phosphorylation sites in Scc1 (Thr^144^ and Thr^312^) match the consensus proposed by Nakajima et al. [24]. These two sites, in addition to one in Scc1 (Ser^454^) and three in SA2 (Thr^1109^, Ser^1137^, and Ser^1224^) conform with the consensus proposed by Barr et al. [25]. These findings are consistent with the possibility that at least some of the sites in Scc1 and SA2 are directly phosphorylated by Plk1.

Alignment of multiple sequences showed that most of the amino acid residues that we found to be phosphorylated in mitotic human Scc1 and SA2 were conserved in orthologs from other vertebrates, but not in more distantly related eukaryotes such as *Drosophila, Caenorhabditis elegans,* or yeast, where the homology to human cohesin subunits is in overall much lower (unpublished data). When we considered the distribution of the sites, we found that Scc1 phosphorylation sites strikingly clustered around the two separase cleavage sites, whereas SA2 phosphorylation sites were concentrated in the C terminus of the protein (Figure 1C). Clustering of Scc1 phosphorylation sites in the vicinity of separase cleavage sites has also been observed in budding yeast [19]. This indicates that, although individual sites on Scc1 are not conserved from yeast to human, the overall pattern of phosphorylation is conserved. Evolutionary conservation of the overall mitotic phosphorylation pattern, but not of individual sites, has also been observed in the case of the anaphase-promoting complex/cyclosome [26].

### Phosphorylation of Scc1 Is Required for Efficient Cleavage by Separase In Vitro

**Figure 2:**
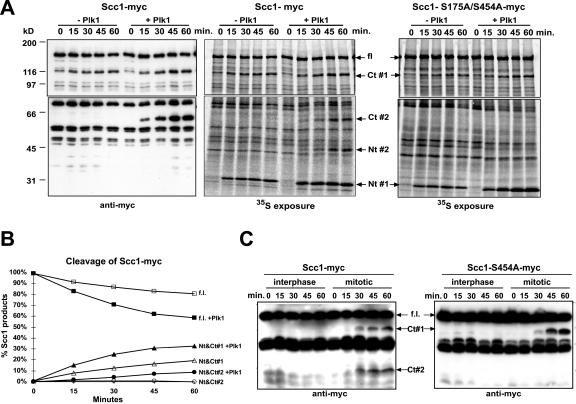
Plk1 Facilitates Cleavage of Human Scc1 by Separase In Vitro (A) Recombinant, ^35^S-labeled, wild-type and mutant Scc1 (see Figure 1C) tagged with 9xmyc at the C terminus were incubated with human separase. Recombinant human GST-Plk1 was added to the reaction mixtures where indicated. Samples were withdrawn from the reactions at the indicated time points and analyzed by SDS-PAGE followed by immunoblotting (anti-myc) and Phosphorimager analysis (^35^S exposure). Arrows indicate full length Scc1-myc (fl), C- and N-terminal fragments resulting from cleavage at Arg^172^ (Ct #1, Nt #1, respectively), and C- and N-terminal fragments resulting from cleavage at Arg^450^ (Ct #2, Nt #2, respectively). The lower parts of the membrane or gels were exposed longer than the upper parts. The enhancement of cleavage at Arg^172^ by Plk1 can be seen particularly well by comparing the intensities of the N-terminal fragments (Nt #1). Note that in the autoradiographs a band can be seen (particularly clearly in the lanes representing the zero time points) that has almost the same electrophoretic mobility as cleavage product Ct #1. This band is distinct from Ct #1 because it migrates a slightly shorter distance and because it is also present in the absence of separase. This band was therefore not included in the quantification in (B). (B) Quantification of the abundance of Scc1-myc and the Scc1-myc cleavage fragments in the assay shown in the left autoradiograph of (A). For the quantification, autoradiographs of identical exposure were used. The sum of the intensities of full-length and all cleavage fragments was set to 100%. Signal intensities for N- and C-terminal fragments resulting from cleavage at the same site were summed. (C) Chromatin fractions were prepared from HeLa cells stably expressing either wild-type Scc1-myc or the mutant Scc1-S^454^A-myc, and were incubated in either interphase or mitotic *Xenopus* egg extract. Mitosis-specific cleavage of Scc1 was detected by immunoblotting with myc antibodies.

In budding yeast, phosphorylation of Scc1 by the Polo-like kinase Cdc5 enhances the rate of cleavage by separase both in vitro and in vivo [19,20]. We therefore analyzed whether treatment of human Scc1 with Plk1 similarly increases its cleavability by separase (Figure 2). We activated purified human separase-securin complexes by securin destruction in mitotic *Xenopus* egg extracts and incubated the activated separase with ^35^S-labeled recombinant Scc1 as a substrate. Under these conditions, separase cleaves Scc1 at the same sites that are cleaved at anaphase onset in vivo, Arg^172^ and Arg^450^ ([15]; Figure 2A). When untreated Scc1 was incubated with separase, efficient cleavage could only be detected at the more N-terminal site, resulting in the formation of one N-terminal and one C-terminal cleavage product which could be seen by Phosphorimager analysis (Figure 2A and 2B). The C-terminal cleavage product could also be detected by immunoblotting using antibodies to a myc-epitope tag on the C-terminus of Scc1 (Figure 2A). When the cleavage reaction was carried out in the presence of active recombinant Plk1, cleavage at the first Scc1 site was slightly enhanced, and cleavage at the second site now became apparent (Figure 2A and 2B), consistent with the possibility that phosphorylation of Scc1 by Plk1 increases the cleavability of Scc1. A cleavage product that corresponds to the fragment in between the two cleavage sites could not be detected under these conditions (Figure 2A), indicating that separase cleaves either one or the other site in one Scc1 molecule, but not or only rarely both.

We suspected that the observed enhancement of Scc1's cleavability in the presence of Plk1 might be due to phosphorylation at two sites that are directly adjacent to the cleavage sites, Ser^175^ and Ser^454^, which we had found to be phosphorylated in mitosis in vivo (Table 1). We therefore mutated these two residues to alanine, thereby creating mutant Scc1-S^175^A/S^454^A (see Figure 1C), and tested the cleavability of this mutant in the absence or presence of Plk1 in vitro. Scc1-S^175^A/S^454^A could still be cleaved at the first site, and this reaction was still slightly enhanced in the presence of Plk1, but cleavage at the second site was completely abolished even in the presence of Plk1 (Figure 2A and 2B). This result suggested that cleavage at Arg^450^ of Scc1 by separase depends on phosphorylation of Ser^454^. The analysis of a Scc1 mutant in which only Ser^454^ was changed to alanine (Scc1-S^454^A) confirmed this notion (unpublished data; see Figure 2C for a related result). Next, we asked whether the enhancement of cleavage at Arg^172^ upon incubation with Plk1 is due to phosphorylation of any of the other residues we had identified. We therefore created a Scc1 mutant in which nine of the ten identified phosphorylation sites were mutated to alanine (Scc1–9xA; see Figure 1C). Mutation of the tenth site was not possible for technical reasons. When Scc1–9xA was incubated with active separase, we found, as expected, that cleavage at Arg^450^ was abolished, but cleavage at Arg^172^ did occur and was still enhanced by the addition of Plk1 (unpublished data). At present, it is therefore unclear how Plk1 enhances cleavage of Scc1 at Arg^172^. The effect could be due to phosphorylation of one or more of the serine and threonine residues at positions 185–189 (Table 1), which were not included in our mutational analysis; alternatively, it might be due to phosphorylation on one or more residues that our mass spectrometry analysis failed to identify. It also remains a formal possibility that Plk1 enhances cleavage at Arg^172^ by phosphorylating separase rather than cohesin.

### Scc1 Phosphorylation Is Not Essential for the Dissociation of Cohesin from Chromosome Arms and for Progression through Mitosis

**Figure 3:**
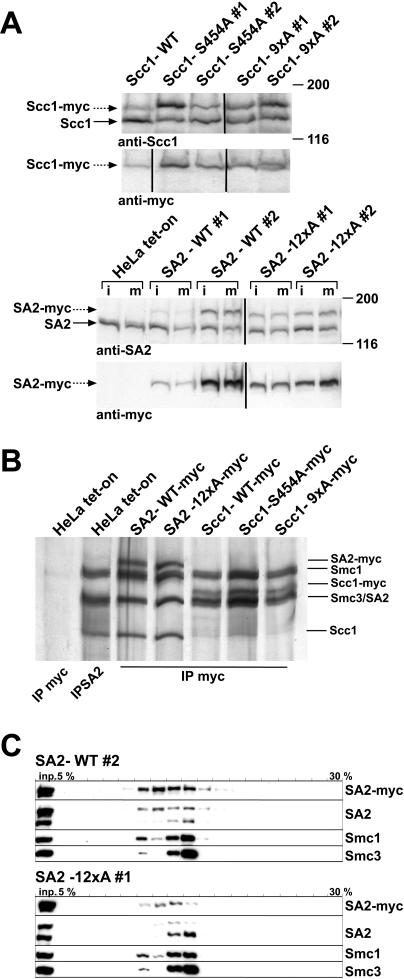
Characterization of HeLa Cell Lines Stably Expressing Wild-Type or Mutant Forms of Human Scc1 and SA2 (A) Wild-type Scc1 or SA2, or the indicated mutant proteins (see Figure 1C), all tagged with 9xmyc at the C terminus, were stably and inducibly expressed in HeLa tet-on cells. After induction by treatment with 2 μg/ml doxycycline for 1–3 d, cell extracts were prepared from either logarithmically proliferating cells (i, interphase) or from cells arrested in mitosis by nocodazole (m, mitosis), then immunoblotted. In the case of Scc1 cell lines (upper blots), only data from interphase extracts are shown. Exogenous protein was detected by immunoblotting with myc antibodies (lower blots). Since the 9xmyc-tag caused a reduced mobility in SDS-PAGE compared to the endogenous protein, Scc1- and SA2-immunoblots (upper blots) revealed the relative amounts of exogenous and endogenous protein in the different cell lines. The position of molecular weight markers is indicated on the right side. (B) Extracts were prepared from the different cell lines as indicated. Immunoprecipitation was performed using myc antibodies, followed by SDS-PAGE and silver staining. As a control, the cohesin complex was immunoprecipitated from untransfected HeLa tet-on cells using antibodies to SA2. (C) Extracts were prepared from SA2-WT-myc or SA2–12xA-myc expressing cells, and fractionated by sucrose density gradient centrifugation (5%–30% sucrose), followed by immunoblotting with antibodies recognizing the proteins indicated on the right (inp. = input/unfractionated sample of the extract).

To address the physiological significance of Scc1 phosphorylation and the resulting enhanced cleavability of Scc1, we generated cell lines that stably express Scc1-S^454^A or Scc1–9xA (Figure 3; see also Figure 1C). To be able to distinguish the ectopically expressed Scc1 proteins from endogenous Scc1, we tagged the Scc1 mutants with nine myc epitopes at their C termini. We have shown that such a tag does not detectably compromise the ability of Scc1 to assemble into cohesin complexes [10] that can establish cohesion [15]. Since expression of mutant Scc1 could have deleterious effects on cells, we furthermore used a doxycycline-regulatable promoter to be able to control the level of expression. To avoid potential overexpression artifacts, we screened by immunoblotting for cell lines in which the ectopically expressed Scc1 is present in amounts that are similar to the amounts of endogenous Scc1 when expression is fully induced by doxycycline (Figure 3A). Immunoprecipitation of the ectopically expressed Scc1 with myc antibodies, followed by SDS-PAGE and silver staining, revealed that the mutated forms of Scc1 could associate with Smc1, Smc3, and SA1/2 into cohesin complexes (Figure 3B; note that endogenous untagged Scc1 does not coimmunoprecipitate with Scc1-myc, indicating that only one Scc1 molecule is present per cohesin complex). Sucrose density gradient centrifugation experiments in conjunction with immunoblotting showed that most of the ectopically expressed Scc1 was incorporated into cohesin complexes (Figure S2A). These observations indicate that any phenotypes (but also the absence of phenotypes) that are observed after ectopic expression of Scc1 are not simply caused by overexpression or by the inability of Scc1 to assemble into cohesin complexes.

To analyze whether mutation of Ser^454^ abolishes cleavage of Scc1 at Arg^450^ also when Scc1 is part of cohesin complexes that have been loaded onto chromatin in vivo, we incubated chromatin from HeLa cells expressing wild-type Scc1, Scc1-S^454^A, or Scc1–9xA in *Xenopus* egg extracts. In this assay, Scc1 is cleaved by separase at Arg^172^ and Arg^450^ if the *Xenopus* egg extract is in a mitotic state [10], presumably because separase and Plx1 are active under these conditions. Whereas wild-type Scc1 was efficiently cleaved at both sites in mitotic egg extract, we did not observe any fragment resulting from cleavage at Arg^450^ when either Scc1-S^454^A or Scc1–9xA was analyzed (Figure 2C and unpublished data), further supporting the conclusion that phosphorylation at Ser^454^ of Scc1 is essential for cleavage at Arg^450^.

Immunofluorescence analysis demonstrated that the localization of Scc1-S^454^A and Scc1–9xA was very similar to the one of wild-type Scc1 (Figure S2B and unpublished data). Both mutants were present in nuclei from telophase through interphase until the next mitosis. In prometaphase, the bulk of mutant cohesin complexes had dissociated from chromosome arms, but small amounts remained at centromeres, even if prometaphase was prolonged by treatment of the cells with nocodazole (Figure S2B). Scc1 phosphorylation at the nine mutated sites is therefore essential neither to load cohesin onto chromatin nor to remove cohesin from chromosome arms in early mitosis. Furthermore, we were unable to observe obvious abnormalities at later stages of mitosis and in the overall ability of cells expressing nonphosphorylatable Scc1 to proliferate (unpublished data), indicating that Scc1 phosphorylation at the mutated sites is not essential for the ability of separase to cleave cohesin complexes and to initiate anaphase. The finding that cells expressing Scc1 in which residue 454 cannot be phosphorylated and in which cleavage at Arg^450^ is therefore compromised (see Figure 2C) do not show anaphase defects is in agreement with our previous observation that Scc1 cleavage at Arg^172^ is sufficient for the viability of HeLa cells [15].

### Phosphorylation of SA2 Is Essential for the Dissociation of Cohesin from Chromosomes during Prophase and Prometaphase

The observation that nonphosphorylatable Scc1 mutants bind chromatin in interphase and dissociate from chromosome arms normally in early mitosis implied that the requirement for Plk1 in cohesin dissociation and the inhibitory effect of cohesin phosphorylation on chromatin binding [16,18] cannot be explained by Scc1 phosphorylation. We therefore asked whether phosphorylation of SA2 might control the association of cohesin with chromosomes. We generated a mutant of SA2 in which serine/threonine residues at 12 sites found to be phosphorylated in mitosis in vivo were mutated to alanine; this mutant was called SA2–12xA (see Figure 1C). We C-terminally tagged this mutant and wild-type SA2 with myc epitopes and expressed both in HeLa cells in a stable and inducible manner, employing the same strategy that we had used for Scc1. Also in this case we isolated cell lines in which the levels of ectopically expressed SA2 are similar to the levels of endogenous SA2 (Figure 3A). Immunoprecipitation experiments with myc antibodies and sucrose density gradient centrifugation showed that both the tagged SA2 wild-type and SA2–12xA proteins were incorporated into cohesin complexes (Figure 3B and 3C).

**Figure 4:**
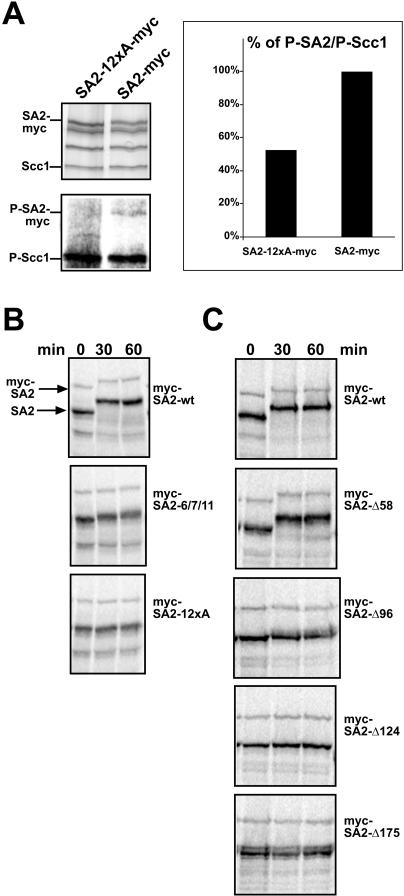
Mutations of the C-Terminal Phosphorylation Sites and C-Terminal Deletions Decrease the Mitotic Phosphorylation of SA2 (A) Cohesin complexes containing wild-type SA2-myc or SA2–12xA-myc were immunopurified by myc antibodies from the respective cell lines. Similar amounts of cohesin were incubated with recombinant GST-Plk1, and the amount of phosphorylation was quantified by ^32^P incorporation followed by SDS-PAGE, silver staining (top) and Phosphorimager analysis, and quantification using the software program ImageJ. (B and C) In vitro-translated, ^35^S-labeled SA2 tagged at the N terminus with 9xmyc was incubated in interphase *Xenopus* egg extracts, which were induced to enter mitosis at time point 0 min by addition of nondegradable cyclin B Δ90 and okadaic acid. Samples were collected at the indicated time points and analyzed by SDS-PAGE followed by Phosphorimager analysis. The autoradiographs in (B) show phosphorylation site mutants and in (C) they show C-terminal deletion mutants (see Figure 1C). The slower-migrating band represents myc-SA2, whereas the faster-migrating band is presumably generated by translation initiation at an internal start codon.

Since SA2 phosphorylation can be catalyzed by purified Plk1 in vitro and depends on Plx1 in *Xenopus* egg extracts [16] we asked whether SA2–12xA had lost the ability to be phosphorylated by Plk1. When purified cohesin complexes were incubated with Plk1 and ^32^P-γ-ATP, approximately 50% less radiolabel was incorporated into SA2–12xA than into wild-type SA2, whereas phosphorylation of Scc1 in the same complexes was not affected (Figure 4A). Because both budding yeast and human Polo-like kinases can phosphorylate many sites in vitro that are not phosphorylated in vivo [26,27], it is possible that the residual phosphorylation of SA2–12xA by Plk1 in vitro occurs on sites that are not phosphorylated in vivo. We therefore analyzed the phosphorylation of wild-type and mutant forms of SA2 under more physiological conditions by incubating ^35^S-labeled recombinant SA2 in mitotic *Xenopus* egg extracts. SA2 phosphorylation in these extracts depends on Plx1 [16] and causes an electrophoretic mobility shift (Figure 4B). This shift was partially abolished when three C-terminal phosphorylation sites were mutated (mutant SA2–6/7/11; see Figure 1C), and no shift could be seen with SA2–12xA (Figure 4B). These results indicate that most mitosis-specific phosphorylation sites have been removed from SA2–12xA. The finding that mitosis-specific phosphorylation sites in SA2 are clustered in the C terminus was also confirmed by generating deletion mutants in which SA2 was progressively shortened from the C terminus (see Figure 1C), because SA2's mobility shift was completely abolished when at least 124 C-terminal residues were removed (Figure 4C).

**Figure 5:**
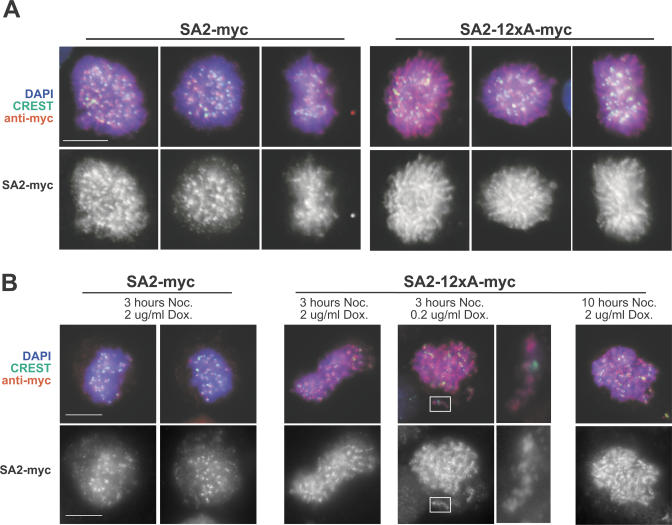
Phosphorylation of SA2 Is Required for Cohesin Dissociation from Chromosome Arms during Prometaphase (A) Logarithmically proliferating HeLa cells expressing SA2-WT-myc or SA2–12xA-myc were extracted prior to fixation, and stained with myc antibodies. In the upper set of images, kinetochores were labeled with human CREST serum, and DNA was counterstained with DAPI. In the lower set of images, only SA2-myc staining is shown. (B) SA2-myc expression was induced by different amounts of doxycycline (Dox.), and cells were arrested in prometaphase by nocodazole (Noc.) treatment for 3 or 10 h. Cells were spun on glass slides, extracted by detergent, fixed, and processed for immunostaining as in (A). Scale bars 10 μm.

To be able to address the physiological relevance of SA2 phosphorylation, we first had to determine whether cohesin complexes containing tagged SA2 behave normally in human cells. Immunofluorescence imaging of wild-type SA2-myc showed a localization pattern that is very similar to the pattern found for Scc1 [10,14,15,18]. SA2-myc was nuclear in interphase, and mainly present in a soluble form in the cytoplasm during mitosis (unpublished data). When we extracted mitotic cells to remove the soluble pool, we found a minor fraction bound to chromatin, and in prometaphase and metaphase, SA2-myc was enriched at centromeres as compared to chromosome arms (Figure 5A). Therefore, the tag in SA2 does not seem to interfere with the behavior of cohesin complexes containing SA2-myc.

When we analyzed the intracellular distribution of the SA2–12xA mutant by immunofluorescence microscopy, we found that this protein was also nuclear throughout interphase and that the nuclear signal could only partially be reduced by removing soluble cohesin complexes by preextraction (unpublished data), indicating that complexes containing SA2–12xA can associate with chromatin like wild-type cohesin. In stark contrast to wild-type SA2, however, SA2–12xA was not strongly enriched at centromeres of prometaphase and metaphase chromosomes, but instead was almost equally abundant on chromosome arms and on centromeres (Figure 5A). A very similar distribution of cohesin on chromosome arms and centromeres has been observed after depletion of Plk1 by RNA interference [18].

These observations indicate that phosphorylation of SA2 is not required for the loading of cohesin onto chromatin, but is essential for its dissociation from chromosome arms during early mitosis. However, it also remained possible that cohesin complexes containing SA2–12xA simply dissociated from chromosomes more slowly than wild-type complexes. To address this possibility, we analyzed cells in which prometaphase was prolonged by treatment with nocodazole. Under these conditions, complexes containing wild-type SA2 dissociated completely from chromosome arms within 3 h but remained at centromeres (Figure 5B), confirming earlier observations made for Scc1 [15,18]. In contrast, SA2–12xA could still be detected clearly on chromosome arms after 3 h and even after 10 h of nocodazole treatment (Figure 5B), excluding the possibility that the dissociation of cohesin complexes containing this mutant is simply slower than the dissociation of wild-type complexes.

### Cohesin Complexes Containing Nonphosphorylatable SA2 Are Able to Establish Cohesion

**Figure 6:**
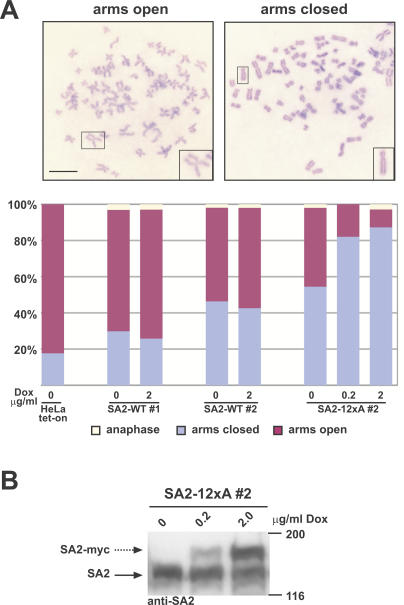
The Presence of SA2–12xA on Chromosome Arms Correlates with Cohesion between Sister Chromatid Arms (A) Cells were cultured in the absence or presence of different amounts of doxycycline as indicated. After arrest in nocodazole for 3 h, cells were fixed, spread on glass slides, and stained with Giemsa (photomicrographs, above). Single chromosomes (indicated by a box) are shown at higher magnification in the lower right corners. The number of cells with chromosome arms that had opened (arms open) or that were connected (arms closed) was scored as indicated (bar graphs, below). Scale bar 10 μm. (B) Whole-cell extracts were prepared from HeLa cells expressing SA2–12xA-myc after treatment with increasing amounts of doxycycline (0, 0.2, and 2.0 μg/ml). The ratio of exogenous SA2–12xA-myc to endogenous SA2 was visualized by immunoblotting with antibodies to SA2. The position of molecular weight markers is indicated on the right.

It was also possible that the amino acid exchanges in SA2–12xA had rendered the protein nonfunctional, possibly resulting in the formation of complexes that bound chromatin nonspecifically and therefore did not dissociate from chromosomes in mitosis. Thus, we asked whether cohesin complexes containing SA2–12xA are able to establish sister chromatid cohesion. To answer this question, we again treated cells with nocodazole, which normally results in the loss of cohesion between chromosome arms but not at centromeres, resulting in the formation of X-shaped chromosomes with “open arms” that can be seen by chromosome spreading and Giemsa staining [18]. In this assay, chromosomes from most cells expressing wild-type SA2-myc showed the normal “open arm” phenotype (between 55% and 71%, depending on the cell line analyzed; Figure 6A). In contrast, only 10% of mitotic cells expressing SA2–12xA contained chromosomes whose arms had lost cohesion during the nocodazole arrest, whereas 87% had maintained cohesion between chromosome arms (Figure 6A). This result strongly indicates that the cohesin complexes that contain SA2–12xA and that remain on chromosome arms in prometaphase (see Figure 5B) are able to establish and maintain cohesion between sister chromatids.

Finally, to further confirm this hypothesis, we asked whether the ability of SA2–12xA-expressing cells to maintain arm cohesion during a nocodazole treatment depends on the amount of ectopic protein that is expressed. We therefore treated cells containing SA2–12xA transgenes with different doses of doxycycline and compared the levels of exogenous SA2 with the arm cohesion phenotype. The levels of mutant SA2-myc were well controlled by the amount of doxycycline used (Figure 6B), and there was a clear correlation between the amount of SA2–12xA and the number of cells whose chromosomes maintained arm cohesion during treatment with nocodazole, whereas expression of wild-type SA2-myc had no significant influence on arm cohesion (Figure 6A). Cells of the SA2–12xA cell line maintained arm cohesion slightly more frequently than control cells even if SA2–12xA expression was not induced, but it is possible that small amounts of the ectopic protein are also synthesized in the absence of doxycycline.

These observations rule out the possibility that the different frequencies with which arm cohesion is observed after nocodazole treatment in SA2–12xA and control cells are due to other differences in the cell lines than expression of the different transgenes. We therefore conclude that cohesin complexes containing SA2–12xA are able to maintain and establish cohesion, but that these complexes are unable to dissociate from chromosomes during prophase and prometaphase. Phosphorylation of SA2 at its C terminus therefore appears to be essential for the unloading of cohesin from chromosome arms during early mitosis.

### Phosphorylation of SA2 Is Required for Efficient Resolution of Sister Chromatids

Although the major amount of human cohesin dissociates from chromosomes during early mitosis, the physiological importance of this process is still unknown. One reasonable assumption is that cohesin dissociation might be required for binding of condensin complexes and for condensation of chromatin. Like cohesin, condensin complexes contain subunits that are members of the Smc and kleisin protein families [28,29,30], and cohesin dissociation and condensin binding normally coincide in cells [3,13]. However, inhibition of cohesin dissociation by interfering with Plk1/Plx1 or Aurora-B function in human cells or *Xenopus* egg extracts did not affect the binding of condensin and the overall condensation of chromosomes [16,17,18,31]. In line with these results, we found that condensin binding, compaction, and shortening of chromosomes in a prolonged mitotic arrest was not detectably influenced by expression of SA2–12xA (Figure S3), further strengthening the notion that condensin binding does not require the phosphorylation-dependent dissociation of cohesin complexes from chromosome arms.

**Figure 7:**
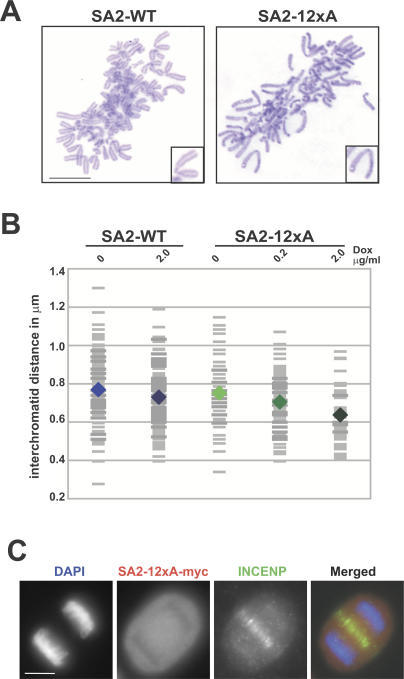
Phosphorylation of SA2 Is Required for Efficient Resolution of Sister Chromatid Arms during Prometaphase and Metaphase (A) HeLa cells expressing SA2-WT-myc or SA2–12xA-myc were spread on glass slides and chromosomes were stained with Giemsa. Representative cells from SA2 WT-myc or SA2–12xA-myc cell lines after induction with 2 μg/ml doxycycline are shown. Scale bar 10 μm. (B) HeLa cells were induced to express SA2-WT-myc or SA2–12xA-myc by different amounts of doxycycline as indicated, and processed as in (A). More than 50 cells in prometaphase or metaphase were selected randomly from each sample. The distance between sister chromatids was determined for five chromosomes in each cell and averaged. Light gray bars indicate average values that have been measured in one or two cells, and darker gray bars indicate average values that have been measured in three or more cells. Diamonds indicate the average distance for all cells in a given sample. (C) Representative immunofluorescence image of normal anaphase in a cell expressing SA2–12xA-myc. The cell was not extracted prior to fixation, so the soluble pool of SA2–12xA-myc is revealed by myc-staining.

Experiments in *Xenopus* egg extracts showed that in the absence of Plx1 and Aurora-B, chromosome arms on mitotic chromosomes remained very close to each other, i.e., the resolution of sister chromatid arms was impaired [17]. In these experiments, it was difficult to distinguish whether Plx1 and Aurora-B promoted sister chromatid resolution by inhibiting the dissociation of cohesin or by another, independent pathway. We therefore examined how chromosome structure is influenced by expression of nonphosphorylatable SA2. We noticed that sister chromatid arms often stayed in closer proximity in SA2–12xA-expressing cells than in controls (Figure 7A). When we measured the interchromatid distance during prometaphase and metaphase, we found a variation within the same cell line from around 0.4 to 1.1 μm between individual cells (Figure 7B). This variability is presumably caused by different periods of time that cells have spent in mitosis; i.e., cells in which the interchromatid distance was small might have spent shorter time in mitosis than those in which sister chromatids were more resolved. We also found that there was some variability between different cell lines (unpublished data). However, cells expressing wild-type SA2 did not show any prominent difference in the average interchromatid distance in the absence or presence of exogenous SA2, whereas the interchromatid distance was progressively shortened when SA2–12xA-myc was expressed in increasing amounts (Figure 7B).

Since resolution of sister chromatid arms happens progressively during prophase and prometaphase, a reduction in the average distance between sister chromatids might also be caused by shortening the time up to metaphase (therefore leaving cells less time to resolve sister chromatids). However, when we compared the percentage of different mitotic stages in SA2–12xA- versus wild-type SA2-expressing cells, we found no indication of a shortening of prometaphase (unpublished data). Phosphorylation of SA2 and dissociation of cohesin from chromosome arms therefore appear to be required for the efficient resolution of sister chromatid arms.

### SA2 Phosphorylation Is Not Essential for Anaphase

The prolonged persistence of cohesin on chromosome arms and the closer proximity of sister chromatid arms might cause defects in anaphase, for example because larger amounts of cohesin cannot be cleaved efficiently enough, or because perturbance of chromosome structure might interfere with the separation of sister chromatids. However, we did not observe obvious anaphase defects in cells expressing nonphosphorylatable SA2. Furthermore, in all anaphases that we observed (*n* > 50), we found that SA2–12xA had completely disappeared from separating chromatids (Figures 7C and S3B). Scc1 cleavage products could be detected in cells expressing SA2–12xA (unpublished data), consistent with the interpretation that the loss of SA2–12xA-containing cohesin complexes from chromatids in anaphase is mediated by separase. These observations indicate that SA2 phosphorylation is required for the dissociation of cohesin from chromosome arms during prophase and prometaphase, but that this dissociation process is not absolutely essential for the initiation of anaphase. These data also further support the notion that separase is able not only to cleave cohesin at centromeres but on chromosome arms as well [18].

## Discussion

It has long been known that cohesin's Scc1 and Scc3/SA1/SA2 subunits are specifically phosphorylated in mitosis [2,5,14,16,19,32,33]. In budding yeast it has been shown that phosphorylation of Scc1 by Cdc5 enhances the cleavability of cohesin by separase [19,20]. In vertebrates, however, the functional significance of these modifications has remained unclear. The circumstantial evidence in vertebrate systems that has existed so far points to a role of cohesin phosphorylation in controlling the ability of cohesin to bind chromatin [2,16], not in modulating Scc1 cleavage by separase. It was also unclear whether Plk1, a kinase that is essential for cohesin dissociation from chromosome arms during prophase and prometaphase [16,17,18], regulates cohesin in early mitosis by directly phosphorylating Scc1 or SA2, or by modifying other proteins that might be required for cohesin unloading.

Our analysis of mitotic cohesin regulation in human cells revealed distinct roles for the phosphorylation of Scc1 and SA2. Phosphorylation of human Scc1 enhances the cleavability of this protein by separase, at least at the second of Scc1's two cleavage sites (see Figure 2), and thereby shows that this mode of cohesin regulation is conserved from yeast to humans. Furthermore, our data imply that Scc1 phosphorylation is not required for the dissociation of cohesin from chromosome arms (see Figure S2), again consistent with the situation in yeast where Scc1 is phosphorylated in mitosis [19], yet cohesin complexes do not dissociate from chromosomes until separase is activated [34,35].

In contrast to the data for Scc1, our results show that SA2 phosphorylation is essential for the dissociation of at least some cohesin complexes from chromosome arms (see Figure 5), but this modification does not seem to be necessary for the cleavage of cohesin complexes by separase (Figure 7C and unpublished data). Cells that express nonphosphorylatable versions of SA2 are unable to remove all cohesin complexes from their chromosome arms during prometaphase (see Figure 5A), even if mitosis is prolonged for many hours by treatment with spindle poisons (see Figure 5B); presumably as a consequence, cohesion between sister chromatid arms is not lost in these cells during prolonged prometaphase arrest (see Figure 6). These phenotypes are virtually identical to the cohesin and cohesion phenotypes of cells in which Plk1 has been depleted [18]. It has also been observed that Plk1 can phosphorylate Scc1 and SA2 in vitro, can thereby decrease the ability of cohesin to bind chromatin, and is required for the mitosis-specific phosphorylation of Scc1 and SA1/SA2 in *Xenopus* egg extracts [16]. Obviously none of these results can exclude the possibility that kinases other than Plk1 contribute to SA2 phosphorylation, nor the possibility that Plk1 may also have to phosphorylate proteins other than SA2 to allow cohesin dissociation, but the simplest interpretation of all the data is that Plk1 is essential for cohesin unloading because it is required for SA2 phosphorylation, which in turn is a prerequisite for cohesin dissociation.

It will be interesting to learn whether this type of regulation is restricted to human SA2, or whether it also applies to paralogs and orthologs of SA2. In addition to SA1 and SA2, a meiosis-specific paralog of the Scc3 family exists in mammals, called SA3 (or STAG3) [36]. Most of the phosphorylation sites that we identified in SA2 are conserved in SA1, whereas SA3 diverges from SA1 and SA2 mostly in its C-terminal sequence (unpublished data). This difference could have important implications for the regulation of meiosis I, where arm cohesion needs to be protected to allow the separation of homologous chromosomes in anaphase I (reviewed in [37]), and where chromosome arms do not separate even if cells are arrested by treatment with spindle poisons [38]. It is possible that the replacement of SA1/2 by SA3 renders meiotic cohesin complexes resistant to Plk1-dependent removal from chromosome arms, and thereby allows the maintenance of arm cohesion until separase is activated. Likewise it will be interesting to analyze whether Scc3 is phosphorylated in budding yeast, in which cohesin dissociation from chromosome arms in early mitosis has not been detected.

### How Important Are Scc1 and SA2 Phosphorylation In Vivo?

Somewhat unexpectedly, we found that expression of neither nonphosphorylatable Scc1 nor nonphosphorylatable SA2 blocked progression through mitosis. Although it remains formally possible that our experiments did not identify all mitosis-specific phosphorylation sites and that we therefore did not mutate all critical sites, we consider it more plausible to think that phosphorylation of these proteins is not absolutely essential for progression through mitosis, at least in transformed cultured cells. This notion is supported by the finding that cohesin can be removed from chromosomes (presumably by separase), even in cells in which Plk1 has been depleted and Aurora-B has been inhibited—i.e., under conditions where the early mitotic dissociation of cohesin from chromosome arms is inhibited [18]. The implication is that the early mitotic dissociation of cohesin from chromosomes is not absolutely essential for mitosis, because separase is able to cleave all cohesin complexes that reside on chromosomes at the metaphase-anaphase transition. In this respect, human cells therefore appear to be more similar to budding yeast than previously suspected, in that HeLa cells can also initiate anaphase without first having to remove cohesin from chromosome arms.

Likewise, there are similarities between yeast and HeLa cells in the regulation of Scc1. In both systems, Scc1 phosphorylation enhances its cleavability by separase, but in neither case is this modification essential for viability (this study) [19]. An interesting hint to the possible function of Scc1 phosphorylation comes from the observation that budding yeast cells lacking the securin Pds1 are viable and are able to undergo anaphase, but this ability is dramatically decreased if phosphorylation sites in Scc1 are mutated [19]. Since securin not only inhibits separase but is also required for its activation, yeast cells lacking securin may not have enough separase activity to cleave cohesin if Scc1 is not phosphorylated. Phosphorylation of Scc1 might increase its affinity for separase, and this effect may simply enhance the fidelity of anaphase initiation. Securin is also not essential for viability in human cells, but in its absence the specific activity of separase is decreased [39]. It would be interesting to test whether human cells lacking securin require Scc1 phosphorylation for viability.

Similarly, it is possible that SA2 phosphorylation and the resulting dissociation of cohesin from chromosomes in early mitosis, albeit not being essential, increase the fidelity of chromosome segregation. It is also conceivable that removal of cohesin prior to cleavage is not important for mitosis but for the next interphase. Separase-dependent cohesin removal destroys the Scc1 subunit and thereby renders cohesin nonfunctional. In contrast, phosphorylation-dependent dissociation appears to leave cohesin intact and might thereby enable the rapid reloading of cohesin onto chromatin in telophase, i.e., without the necessity for new Scc1 transcription and translation, which is inhibited during mitosis.

### How Does SA2 Phosphorylation Lead to Cohesin Dissociation?

Cohesin is bound to chromatin in an extremely stable manner ([8]; E.R. and J.M.P, unpublished data), and this may be related to the fact that Smc1, Smc3, and Scc1 form a ring-like complex, at least in budding yeast [40,41]. It has been proposed that this protein ring establishes cohesion by encircling the sister chromatid strands [40]. In this model, it is easy to imagine how cleavage of Scc1 releases cohesin from chromatin. However, Scc3 binds to Scc1 and is not required for formation of the ring-like complex, and it is therefore not immediately obvious how phosphorylation of SA2 could lead to dissociation of cohesin from chromosomes. One possibility is that SA2 phosphorylation induces a conformational change in cohesin that opens the ring. Bulk phosphorylation of SA2's C terminus, for example, might considerably change its surface charge, thereby affecting interactions between Scc1 and the Smc1/3 subunits. In its simplest form, this model would predict that SA2 phosphorylation is sufficient for opening of the cohesin ring and thus is sufficient for cohesin dissociation. However, in preliminary experiments, we have been unable to observe cohesin dissociation when we added purified active Plk1 to chromatin (I. Sumara and J.M.P, unpublished data), whereas the simultaneous addition of Plk1 and *Xenopus* egg extracts to chromatin did enable cohesin dissociation [16]. It is therefore also possible that phosphorylation of SA2 recruits cohesin unloading factors to chromatin (which in the above experiment might have been contributed by the *Xenopus* extract), which then somehow enable the dissociation of cohesin from chromosomes. In budding yeast and *C. elegans,* cohesin needs additional factors for its loading onto chromatin [42,43]. Cohesin might similarly need aid for unloading, at least in the absence of Scc1 cleavage. If such additional factors exist and interact with SA2, the cell lines we created might provide a means to isolate the relevant molecules by differential purification of cohesin complexes containing wild-type SA2 and SA2–12xA.

If SA2 phosphorylation results in cohesin unloading by somehow enabling the opening of the cohesin ring without its cleavage, it would be conceivable that this reaction is simply the reverse of the loading process, during which the cohesin ring presumably also has to be opened transiently [44]. However, a prediction of this model would be that SA2 phosphorylation would also be required for the loading of the cohesin complex, whereas we find that complexes containing nonphosphorylatable cohesin can efficiently associate with chromatin and even establish functional cohesion. It is therefore more plausible to hypothesize that SA2 phosphorylation is a modification that is specifically used to remove cohesin from chromosomes in early mitosis by enabling a reaction that is not simply the reverse of the loading reaction.

### Which Cohesin Complexes Are Regulated by SA2 Phosphorylation?

SA2 phosphorylation is required for the dissociation of cohesin from chromosome arms, but it does not seem to affect the behavior of cohesin at centromeres. As a consequence, sister chromatid arms are resolved much farther from each other than centromeres during a normal prometaphase, and they can lose cohesion completely if prometaphase is prolonged, whereas cohesion at centromeres is protected. How is this regulation achieved? Recent work in fission yeast has shown that members of the Mei-S332 family of proteins [45] are required for the persistence of cohesin at centromeres in meiosis I [46,47,48,49]. These proteins, called shugoshins or Sgo1, are thought to protect centromeric cohesin in anaphase I from premature cleavage by separase, but they are also found at centromeres in mitotic *Drosophila* and budding yeast cells [49,50]. In an associated paper by McGuinness et al. [51], we show that an ortholog of Sgo1 is also required for the persistence of cohesin at centromeres and for the maintenance of sister chromatid cohesion during prometaphase in human cells. Remarkably, Sgo1-depleted cells do not show cohesion defects if a nonphosphorylatable form of SA2 is expressed. This observation implies that cohesin normally persists at centromeres because Sgo1 protects cohesin in this chromosomal domain from phosphorylation. To test this hypothesis it will be important to determine whether SA2 phosphorylation does occur at centromeres. Our identification of in vivo phosphorylation sites on SA2 may be an important prerequisite for achieving this goal, because it should enable the generation of phosphospecific antibodies. Likewise, it will be interesting to learn how Sgo1 prevents or antagonizes SA2 phosphorylation, and whether the same mechanism is able to protect centromeric cohesin from separase in meiosis I.

Previous work has highlighted the difference in the regulation of cohesin complexes between chromosome arms and centromeres [10,18], but several observations suggest that there may also be important differences among different populations of cohesin complexes on chromosome arms. When we compared Scc1 cleavage in cells expressing either wild-type or nonphosphorylatable SA2, we noticed that the levels of Scc1 cleavage products in the latter cells were only slightly increased, if at all (unpublished data). If all complexes containing nonphosphorylatable SA2 remained on chromosome arms until prometaphase, we would instead expect to see more Scc1 cleavage in cells containing these complexes than in cells containing wild-type SA2. Furthermore, we noticed that the immunofluorescence intensity of SA2–12xA-myc in interphase cells from which soluble cohesin complexes had been removed by preextraction was clearly higher than the intensity of SA2–12xA-myc on prometaphase and metaphase chromosomes, and in subcellular fractionation and immunoblotting experiments we found that a fraction of SA2–12xA still became soluble in nocodazole-arrested cells (unpublished data). We made similar observations in immunofluorescence experiments in which we analyzed the chromosome association of Scc1-myc in Plk1-depleted cells. Also in these cells, some cohesin still seemed to dissociate from chromosome arms, despite the depletion of Plk1 (T. Hirota and J.M.P, unpublished data). The observation that some cohesin complexes do dissociate from chromosome arms even if Plk1 is depleted or if these complexes contain nonphosphorylatable SA2, whereas others do not, cannot simply be explained by slow dissociation kinetics of cohesin under these conditions, because those complexes that persist on chromosome arms can still be found there after 10 h of prometaphase arrest (see Figure 5B). We therefore favor the hypothesis that there are, in fact, two distinct populations of cohesin on chromosome arms: one whose dissociation depends on Plk1 activity and SA2 phosphorylation, and one whose dissociation does not. Since the population of cohesin complexes whose dissociation depends on Plk1 and SA2 is able to establish cohesion (see Figure 6), it is possible that those complexes that seem to be able to dissociate without Plk1 and SA2 phosphorylation are bound to chromatin in a manner that does not establish cohesion. Such binding modes must exist, because cohesin rebinds to chromatin in telophase [3,13], i.e., long before sister chromatids have been generated by DNA replication. This speculative model makes important predictions, for example that cohesin dissociation from unreplicated DNA should not depend on SA2 phosphorylation; we will attempt to test this prediction in the future.

## Materials and Methods

### 

#### Cell lines and growth conditions

HeLa cells expressing myc-tagged human Scc1 were described previously [15]. HeLa cells expressing mutated Scc1, wild-type SA2, or mutated SA2 were generated by transfecting HeLa tet-on cells (Clontech, Palo Alto, California, United States) with the pTRE2-hygro vector (Clontech) containing the respective cDNA and a 9xmyc tag fused in frame. Hygromycin-resistant clones were selected by growth in medium containing 400 μg/ml hygromycin, and were tested for expression of the myc-tagged protein by immunoblotting after induction with 2 μg/ml doxycycline for 1–3 d. Untransfected HeLa cells were grown in DMEM supplemented with 10% FCS, 0.2 mM L-glutamine, 100 U/ml penicillin, and 100 μg/ml streptomycin. For growth of stably transfected HeLa tet-on cells, the medium was supplemented with 200 μg/ml G418 and 100 μg/ml hygromycin. Hydroxyurea and nocodazole were used at concentrations of 2 mM and 330 nM, respectively.

#### cDNA mutagenesis

Serine or threonine residues in human Scc1 and SA2 were changed to alanine by mutation of the respective cDNAs using the Quick Change Site-Directed or Multi Site-Directed Mutagenesis kit (Stratagene, La Jolla, California, United States). Where the phosphorylated site could not be assigned to a certain residue within a peptide, we mutated all possible candidate sites to alanine residues. C-terminally truncated versions of SA2 were generated by PCR. For in vitro translation, cDNAs were cloned into pcDNA 3.1 (−)/Myc-His A (Invitrogen) modified to contain a 9xmyc cassette. The expression of cDNAs as ^35^S-methionine- and ^35^S-cysteine-labeled proteins was performed using a coupled transcription-translation system in rabbit reticulocyte lysate (Promega, Madison, Wisconsin, United States).

#### Antibodies

Antibodies specific to phosphorylated threonine were from Cell Signaling Technology (Beverly, Massachusetts, United States; #9381). To detect myc-tagged protein, we used either polyclonal, affinity-purified rabbit anti-myc antibody (Gramsch Laboratories, Schwabhausen, Germany; #CM-100), or monoclonal 9E10 mouse-anti-myc antibody [10]. Polyclonal antibodies to human Scc1, SA1, SA2, Smc1, Smc3, and Smc2 have been described [3,10,30]. Anti-INCENP peptide antibody was raised in rabbit, and the serum was affinity purified. The following peptide was used for immunization: TDQADGPREPPQSARRKRSYC. Human CREST serum was a kind gift of A. Kromminga (Institut für Immunologie, Pathologie und Molekularbiologie, Hamburg, Germany).

#### Protein purification and fractionation

Endogenous cohesin was purified from HeLa cell extracts by immunoprecipitation using purified SA2 peptide antibodies (antibody 447) crosslinked to Affi-Prep protein A beads (Bio-Rad, Hercules, California, United States) as described [3,16]. For mass spectrometric analysis, cohesin precipitates were first washed with IP buffer (20 mM Tris-HCl [pH 7.5], 100 mM NaCl, 0.2% Nonidet P-40, 20 mM β-glycerophosphate, 10% glycerol, 1 mM NaF, and 0.5 mM DTT) [3] supplemented with 400 mM NaCl, followed by a wash step with IP buffer not containing detergent. Cohesin was eluted from the antibody beads by addition of 1.5 bead volumes of 100 mM glycine (pH 2.2). The samples were titrated to pH 8.5 by addition of NH_4_HCO_3_ to a final concentration of 200 mM. Cohesin complexes containing myc-tagged subunits were immunopurified with rabbit anti-myc antibodies (Gramsch Laboratories, #CM-100) using similar conditions. Sucrose density gradient centrifugation was performed as described [15].

#### Mass spectrometry

A volume of 100 μl of immunopurified cohesin (isolated from about 10 mg of total HeLa protein) was reduced with 1 μg of DTT for 1 h at 37 °C and alkylated with 5 μg of iodoacetamide for 30 min at room temperature in the dark. The proteins were digested in solution with one of the following proteases or combination of proteases: 200 ng of trypsin for 4 h at 37 °C, followed by addition of another 200 ng of trypsin and further incubation for 4 h at 37 °C; 400 ng of Glu-C for 8 h at 25 °C; 400 ng of trypsin overnight at 37 °C followed by 400 ng of Glu-C for 8 h at 25 °C; 400 ng of chymotrypsin or elastase for 4 h at 25 °C (all proteases were sequencing grade; Roche, Basel, Switzerland); or 300 ng of subtilisin (Fluka, from Sigma-Aldrich, St. Louis, Missouri, United States) for 30 min at 37 °C. Generated peptides (100-μl sample) were separated by reversed phase nano-HPLC (LC Packings, Sunnyvale, California, United States) and analyzed using an ion trap mass spectrometer (ThermoFinnigan, from Thermo Electric, Waltham, Massachusetts, United States) as described by Mitulovic et al. [52]. All tandem mass spectra were searched against the human nonredundant protein database by using the SEQUEST program (ThermoFinnigan). Any phosphopeptide matched by computer searching algorithms was verified manually.

#### In vitro Scc1 cleavage assays

In vitro cleavage assays were performed as described [10] with the exception that for the assays in Figure 2A, in vitro-translated human wild-type or mutant Scc1-myc was used as substrate. To isolate human separase, purified polyclonal antibodies generated against recombinant human separase were used (kindly provided by I. Waizenegger). In some reactions human GST-Plk1 (16) was added in a concentration of approximately 50 ng/μl.

#### SA2 electrophoretic mobility shift assays

*Xenopus* interphase egg extracts were supplemented with 1/20 volume of in vitro-translated ^35^S-labeled SA2, which had C-terminal deletions or mutations of phosphorylation sites. All SA2 constructs used in this assay lacked the 69 N-terminal amino acids, because the start codon was initially misassigned. Extracts were induced to enter mitosis by addition of cyclin B Δ90 and 1 μM okadaic acid as described in Sumara et al. [3]. Samples were collected at the indicated time points and analyzed by SDS-PAGE followed by Phosphorimager analysis (Storm, Amersham Biosciences, Little Chalfont, United Kingdom).

#### In vitro phosphorylation assay

SA2-myc or SA2–12xA-myc containing cohesin complexes were purified by immunoprecipitation with affinity-purified rabbit anti-myc antibody (Gramsch Laboratories, # CM-100). Conditions of the in vitro phosphorylation assay have been described [26].

#### Microscopy

For immunofluorescence microscopy, cells were either grown on coverslips or spun onto glass slides using a Cytospin centrifuge (Shandon brand, available from Thermo Electric). Cells were extracted with 0.1% Triton X-100 prior to fixation to remove the soluble pool of cohesin. Fixation, immunostaining, and image acquisition were performed as described [10]. Chromosome spreads followed by Giemsa staining were performed as described [31].

#### Quantification of interchromatid distance

On pictures of chromosome spreads, a line scan was performed across chromosome arms orthogonal to the long axis of the chromosome using MetaMorph software (Universal Imaging, Downingtown, Pennsylvania, United States). On the line scan, the distance from peak to peak was measured. Five chromosomes were thus analyzed per cell, and the resulting distances were averaged. More than 50 cells were randomly picked per cell line, and thus analyzed.

## Supporting Information

Figure S1 Phosphorylation Sites in Human Scc1 and SA2 Sites for Scc1 (A) and SA2 (B) are shown. The sites marked in red and blue were found to be phosphorylated in mitosis by mass spectrometry. Residues marked in red were unambiguously identified, whereas in the regions marked in blue the phosphorylated residue could not be assigned with certainty. The separase recognition sites on human Scc1 (A) are indicated in green. Peptides identified by mass spectrometry are highlighted in yellow. The residues at which SA2 was truncated for the assay in Figure 4C are also indicated (B).
(1.2 MB EPS).

Figure S2 Phosphorylation of Scc1 Is Not Required for Dissociation of Cohesin from Chromosome Arms during Prometaphase and Metaphase (A) Extracts were prepared from HeLa cells expressing Scc1-S^454^A-myc or Scc1–9xA-myc and fractionated by sucrose density gradient centrifugation (5%–30% sucrose), followed by immunoblotting with antibodies recognizing the proteins indicated on the right (inp. = input/unfractionated sample of the extract).
(B) HeLa cells expressing Scc1 WT-myc or Scc1–9xA-myc were either grown logarithmically (0 h Noc) or arrested in prometaphase for 5 h by nocodazole (5 h Noc). Cells were extracted prior to fixation, and stained with myc-antibodies. Kinetochores were labeled with human CREST (calcinosis, Raynaud phenomenon, esophageal dysmotility, sclerodactyly, telangiectasias) serum, and DNA was counterstained with DAPI. Scale bar, 10 μm.
(870 KB JPG).

Figure S3 Condensation or Condensin Binding Is Not Impaired in SA2–12xA-Expressing Cells (A) Untransfected HeLa tet-on cells and HeLa cells expressing SA2-WT-myc, or SA2–12xA-myc were arrested with nocodazole for 10 h. Cells were fixed, spread on glass slides, and stained with Giemsa. For each sample, one representative cell is shown. The small bars next to one of the chromosomes in all panels have the same length.
(B) HeLa cells expressing SA2-WT-myc or SA2–12xA-myc were spread on glass slides, extracted prior to fixation, and immunostained as indicated, using an antibody against human Smc2 to reveal condensin. Scale bars in (A) and (B), 10 μm.
(721 KB JPG).

### Accession Numbers

GenBank accession numbers for proteins discussed in this paper are human Scc1 (NP_006256) and human SA2 (NP_006594).
